# Recent clinical advances in fibroblast activation protein targeted radioligand therapy for solid tumors

**DOI:** 10.3389/fnume.2025.1737443

**Published:** 2025-12-05

**Authors:** Xiaolong Wu, Yu Wang, Yingchun Li, Wenli Hu

**Affiliations:** 1 Thoracic Oncology Ward, Cancer Center, and State Key Laboratory of Biotherapy, West China Hospital, Sichuan University, Chengdu, China; 2 Department of Nuclear Medicine and Radiation Oncology, Air Force Hospital Medical Service Department in Western Theatre, Chengdu, China; 3 Department of Pharmacy, Air Force Hospital Medical Service Department in Western Theatre, Chengdu, China

**Keywords:** fibroblast activation protein, FAP, FAP inhibitor, FAPI, radioligand therapy, solid tumors

## Abstract

Fibroblast activation protein (FAP) is a type II transmembrane serine protease predominantly expressed by cancer-associated fibroblasts (CAFs) in more than 90% of epithelial malignancies. The advent of FAP inhibitor (FAPI)-based positron emission tomography (PET) imaging has established FAP as a promising pan-tumor target for radioligand therapy (RLT). This review summarizes the current clinical landscape of FAP-targeted RLT in solid tumors, while also discussing existing challenges and future directions in this rapidly evolving field.

## Introduction

Fibroblast activation protein (FAP) is markedly overexpressed within the tumor stroma of a wide range of malignancies, representing a potential target for both molecular imaging and therapeutic intervention (1, 2). Despite extensive research over the past two decades, most FAP-targeted therapeutic approaches have shown limited clinical translation, with the majority remaining in preclinical development (3). The emergence of FAP inhibitor (FAPI)-PET imaging has significantly accelerated progress in FAP-targeted radioligand therapy (FAP-RLT) (2, 4). This review provides an overview of recent clinical advances in FAP-RLT for solid tumors and identifies key research gaps and prospective directions for future investigation.

## Overview of FAP-RLT

Among the various FAP-targeted therapeutic approaches (e.g., small-molecule enzyme inhibitors, monoclonal antibodies, vaccines, prodrugs, and chimeric antigen receptor T cells), FAP-RLT currently represents the most clinically advanced strategy. This progress is largely attributable to the successful application of FAP-targeted PET imaging, which enables effective visualization of a wide range of solid tumors (2, 4). Although FAP-PET imaging has certain limitations (e.g., overlapped uptake between tumor and benign fibrotic lesions), its noninvasive nature provides a valuable tool for both identifying suitable candidates for FAP-RLT and monitoring therapeutic response. By replacing the imaging isotope with a therapeutic isotope, FAP-targeted ligand can be used as a therapeutic agent. At present, several FAP-targeting radiopharmaceuticals labeled with β-emitting isotopes (^90^Y, ^177^Lu or ^153^Sm) or *α*-emitter isotopes (^213^Bi or ^225^Ac) are undergoing evaluation in early-phase clinical trials. Notable agents in development include FAPI-46, (SA.FAPi)_2_, EB-FAPI, FAP-2286, 3BP-3940, and FAPI-RGD. Preliminary clinical data indicate that these compounds are generally well-tolerated, with some demonstrating encouraging antitumor activity (Table 1).

**Table 1:** Clinical trials of FAP-RLT.

Agent	Isotope	Conditions	Phase	Sample size	Results	Status	NCT	Ref.
FAPI-46	^90^Y	Advanced solid tumors	N/A	21	1 PR, 7 SD	Discontinued	N/A	(5)
FAPI-46	^90^Y	STF	Case study	11	82% DCR, 3 PR, 6 SD, mPFS 227 days	N/A	N/A	(6)
FAPI-46	^90^Y	STF	Case study	3	2 PR, 1 SD, 1 PMR, 1 CMR	N/A	N/A	(7)
FAPI-46	^213^Bi	Metastatic solid tumors	Case study	6	1 PR, 1 SD, 4 PD	N/A	N/A	(10)
DOTAGA. (SA.FAPi)_2_	^177^Lu	RR-DTC	N/A	15	4 PR, 3 SD	N/A	N/A	(14)
DOTAGA. (SA.FAPi)_2_	^177^Lu	BC	N/A	19	25% PR, 37.5% PD, mOS 12 mos, mPFS 8.5 mos	N/A	N/A	(15)
DOTAGA. (SA.FAPi)_2_	^177^Lu	RAIR TC	N/A	36	50% PR, 25% SD, 25% PD, mOS 32 mos, mPFS 29 mos	N/A	N/A	(16)
EB-FAPI	^177^Lu	RAIR TC	I	12	25% ORR, 83% DCR, 25% PR, 58% SD, 17% PD	Completed	NCT05410821	(21)
EB-FAPI	^177^Lu	Advanced solid tumors	II	28	46% DCR, 4 PR, 9 SD	Completed	NCT05963386	(22)
FAP-2286	^177^Lu	Advanced solid tumors	N/A	11	2 SD, 9 PD, well tolerated	N/A	N/A	(26)
FAP-2286	^177^Lu	Advanced solid tumors	I/II	222	1 PR, 9 SD among 27 pts in phase I	Recruiting	NCT04939610	(27)
FAP-2286	^177^Lu	Advanced sarcoma	Case study	5	4 PR, 1 PD	Completed	N/A	(28)
FAP-2286	^177^Lu	Advanced lung cancer	Case study	9	78% ORR, 44% PR, 33.3% SD, 22.2% PD, mOS 10 mos, mPFS 6 mos	Completed	N/A	(29)
3BP-3940	^177^Lu-/^90^Y-/^225^Ac	Advanced solid tumors	N/A	88	66.7% ORR, 80.4% DCR, 2 CR, 32 PR, 7 SD, 8 PD, 2 MR among 51 evaluable pts	N/A	N/A	(32)
FAPI-RGD	^177^Lu	Advanced solid tumors	I	15	2 PR, 6 SD, 1 PD for the first 9 pts enrolled	Recruiting	NCT06638034	(37)
PNT6555	^177^Lu	Advanced solid tumors	I	20	Limited tumor retention	Terminated	NCT05432193	(39)
XT117	^177^Lu	Advanced solid tumors	I	20	Ongoing	Recruiting	NCT06197139	
XT117	^177^Lu	Advanced solid tumors	I	20	Ongoing	Recruiting	NCT06211647	
JH04	^177^Lu	FAP-positive tumors	I	9	Ongoing	Recruiting	NCT06636617	
FAPI-05	^177^Lu	Advanced solid tumors	I	50	Ongoing	Not yet recruiting	NCT06553846	
OncoFAP-23	^177^Lu	FAP-positive tumors	I	56	Ongoing	Not yet recruiting	NCT06640413	
EB-FAPI	^177^Lu	Advanced cholopancreatic tumors	I	29	Unknown	Unknown	NCT06081322	
NNS309V	^177^Lu	Advanced solid tumors	I	124	Ongoing	Recruiting	NCT06562192	
DOTA-FAPI	^177^Lu	Advanced solid tumors	I	30	Unknown	Unknown	NCT04849247	

FAP, fibroblast activation protein; FAPI, fibroblast activation protein inhibitor; EB, Evans blue; mOS, median overall survival; mPFS, median progression free survival; ORR, objective response rate; DCR, disease control rate; CR, complete response; PR, partial response; CMR, complete metabolic response; PMR, partial metabolic response; MR, minor response; SD, stable disease; PD, progressive disease; pts, patients; STF, solitary fibrous tumor; BC, breast cancer; RR-DTC, radioiodine-refractory differentiated thyroid carcinoma; RAIR TC, radioiodine-refractory thyroid cancer; N/A, not available.

### FAPI-46

As the biological half-life of FAPI-46 is relatively short (24 h), it is better aligned with isotypes with similar half-life, such as ^90^Y (64 h) to get optimal radiation dose delivery to the target tissue. Fendler et al. administered up to four cycles of [^90^Y]Y-FAPI-46 to a cohort of 21 patients with advanced solid malignancies (5). Treatment-related grade 3 or 4 adverse events were observed in 8 patients (38%), with thrombocytopenia and anemia being the most frequently reported toxicities. Among the 16 evaluable patients, response assessment according to PERCIST criteria revealed disease control in 8 patients (50%), comprising 1 partial response (PR) and 7 stable disease (SD) (5). Moreover, median overall survival (OS) was significantly longer for RECIST responders, stratified by response category: partial response (not reached), stable disease (14.4 months), progressive disease (6.6 months), nonavailable response status (2.2 months). Notably, the observed clinical benefits (PR and SD) were predominantly confined to patients with sarcoma, particularly those with solitary fibrous tumor (SFT) (5). Based on these findings, a subsequent study by the same group focused exclusively on SFT, where immunohistochemistry demonstrated FAP expression predominantly localized to tumor cell membranes. In this follow-up investigation, 11 patients with SFT received a total of 34 cycles of [^90^Y]Y-FAPI-46 (median: 3 cycles per patient), resulting in disease control in 9 patients (82%), including 3 PR and 6 SD, and a median progression-free survival (PFS) of 227 days (6). In a recent case report study, 3 patients with metastatic SFT who were exhausted standard treatments received 4 cycles of [^90^Y]Y-FAPI-46 with a cumulative dose of approximately 26 GBq (7). Treatment was well tolerated, with only minor adverse events observed. RECIST assessment indicated 2 PR and 1 SD. Of note, PERCIST assessment revealed one patient with a complete metabolic response, with complete resolution of ^18^F-FDG uptake in measurable target lesions (7).

However, the biological half-life of FAPI-46 (24 h) is still shorter than that of ^90^Y (64 h), limiting optimal radiation dose delivery to the target tissue. To better match the pharmacokinetics of FAPI-46, Kratochwil et al. labeled FAPI-46 with short physical half-life (46.3 h) ^153^Sm and tested its feasibility in a patient with metastatic soft tissue sarcoma (8). Emission scans during therapy demonstrated tumor targeting up to 44 h p.i. and rapid clearance from normal organs. Three cycles with cumulative 20 GBq [^153^Sm]Sm- and 8 GBq [^90^Y]Y-FAPI-46 were well tolerated and achieved stable disease for 8 months (8). However, the utilization of ^153^Sm is challenging so far as it is inevitably contaminated with ^154^Eu during manufacturing process (9). An alternative option is to conjugate FAPI-46 with ^213^Bi, an α-emitter with a half-life of 46 min. Recently, Helisch et al. conducted a pilot study of [^213^Bi]Bi-FAPI-46 in 6 patients with progressive metastatic solid tumors (10). A mean of 1,609 MBq of [^213^Bi]Bi-FAPI-46, fractionated into 53 single administrations (range, 5–12 RLT administrations per patient) was well tolerated without adverse side effects, resulting in 1 PR, 1 SD and 4 progressive disease (PD). This study demonstrated that fractionated [^213^Bi]Bi-FAPI-46 is a promising approach that matches the pharmacokinetics of FAPI-46 better than the ^177^Lu- or ^90^Y-labeled compounds (10).

### DOTAGA (SA.FAPi)_2_

To enhance tumor retention, Moon et al. (11) and Martin et al. (12) developed bivalent FAP inhibitors such as DOTAGA.(SA.FAPi)₂, which exhibited prolonged tumor residence time and increased uptake compared to the monomeric DOTA.SA.FAPi (effective half-life, 86.6 h vs. 14 h) (13). [^177^Lu]Lu-DOTAGA.(SA.FAPi)_2_ demonstrated effective half-lives of 46.2 h and 86.6 h in the whole body and tumor lesions, respectively (13). In a prospective exploratory study involving 15 patients with radioiodine-refractory differentiated thyroid carcinoma (RR-DTC), administration of [^177^Lu]Lu-DOTAGA.(SA.FAPi)_2_ was well tolerated, with no reported grade 3/4 hematologic, renal, or hepatic toxicities. Molecular imaging-based response assessment revealed PR in 4 patients and SD in 3 patients (14).

A retrospective analysis was subsequently conducted in 19 patients with metastatic breast cancer who received 2–6 cycles of [^177^Lu]Lu-DOTAGA.(SA.FAPi)_2_ (15). Treatment was well tolerated, with no grade ≥3 adverse events. Response assessment using PERCIST criteria via [^68^Ga]Ga-DOTA.SA.FAPi PET/CT revealed PR in 25% of patients and PD in 37.5%, with an overall clinical benefit rate of 84%. According to the visual analog score (VAS), 26.3% of patients achieved complete response (CR), 15.7% PR, 42% minimal response, 11% SD, and 5% exhibited no response. At the time of analysis, median overall survival (OS) was 12 months, and median PFS was 8.5 months (15). More recently, long-term outcomes of [^177^Lu]Lu-DOTAGA.(SA.FAPi)_2_ therapy in patients with radioiodine-resistant follicular cell-derived thyroid cancer were reported in another retrospective study (16). A median cumulative activity of 22.2 GBq (range: 4–55.5 GBq) was administered over 1–9 treatment cycles (median: 3 cycles). Four patients (5.4%) encountered grade 3 anemia, primarily linked to bone metastasis in three cases and neck tumor mass bleed in one. Grade 3 thrombocytopenia occurred in three patients (4%). No grade ≥ 3 renal or hepatic toxicity was reported. Among 36 evaluable patients, PERCIST-based molecular response assessment demonstrated PR in 50%, SD in 25%, and PD in 25%. Median OS and PFS were 32 months and 29 months, respectively (16).

### EB-FAPI

Serum albumin functions as a highly versatile endogenous carrier for therapeutic agents, enabling reversible binding with drug molecules to form albumin-drug complexes that serve as circulating reservoirs. This interaction enhances both systemic distribution and bioavailability of the therapeutic payload (17). To exploit this mechanism, Wen and colleagues developed [^177^Lu]Lu-EB-FAPI (designated as [^177^Lu]Lu-LNC1004) by conjugating FAPI-02 with Evans blue (EB) (18), an albumin-binding moiety known for its strong affinity toward binding site 1 on serum albumin (19, 20). This modification significantly improved tumor uptake and retention, with sustained tumor accumulation observed up to 96 h post-injection (18). In a first-in-human dose-escalation study involving 12 patients with metastatic radioiodine-refractory thyroid cancer (mRAIR-TC), 3.33 GBq/cycle of [^177^Lu]Lu-LNC1004 was well tolerated with one patient experienced grade 4 thrombocytopenia and three with no dose-limiting toxicities (21). Dose escalation to 4.99 GBq per cycle resulted in grade 3 and 4 hematologic toxicities in two patients. The effective half-lives of [^177^Lu]Lu-LNC1004 were estimated at 90.20 h for whole-body and 92.46 h within tumor lesions. Based on RECIST v1.1 criteria, PR, SD, and PD were observed in 3 (25%), 7 (58%), and 2 (17%) patients, respectively, resulting in an objective response rate (ORR) of 25% and disease control rate (DCR) of 83% (21).

Subsequently, in a phase II investigator-initiated trial, 28 patients with progressive metastatic solid tumors received 2–4 cycles of [^177^Lu]Lu-LNC1004 at a fixed dose of 3.33 GBq every six weeks (22). The mean absorbed radiation dose to tumors was 4.69 ± 3.83 Gy/GBq (range: 1.18–25.03 Gy/GBq). Treatment-related grade 3/4 hematotoxicities occurred in 21% (*n* = 6) of patients, predominantly involving thrombocytopenia, leukopenia, and neutropenia. No grade ≥ 3 hepatotoxicity or nephrotoxicity was observed. Disease control as per RECIST criteria was achieved in 13 of 28 patients (46%), including 4 PR and 9 SD, which was significantly associated with prolonged PFS and OS (*P* < 0.001) (22).

### FAP-2286

FAP-2286 is a cyclic FAP-binding peptide linked to DOTA, allowing radionuclide chelation for imaging or therapeutic applications (23–25). In preclinical HEK-FAP mouse models, [^177^Lu]Lu-FAP-2286 demonstrated prolonged tumor retention time (≥ 72 h) compared to FAPI-46 (24 h) after injection (23). In its first-in-human trial for treatment of 11 patients with advanced solid tumors, [^177^Lu]Lu-FAP-2286 was administrated at a mean dose of 5.8 ± 2.0 GBq per cycle (26). The treatment was well tolerated, with prolonged tumor retention observed for up to 10 days post-injection. The effective half-lives of [^177^Lu]Lu-FAP-2286 were estimated at 35 h for whole body and 44 h for bone metastases. No grade 4 adverse events were reported. Grade 3 adverse events occurred in three patients, comprising pancytopenia (*n* = 1), leukocytopenia (*n* = 1), and pain flare (*n* = 1). Notably, three patients reported symptomatic pain relief following therapy. According to RECIST 1.1 criteria, two patients achieved SD, while the remaining nine experienced PD (26).

Building upon these findings, a phase I/II clinical trial (LuMIERE study; NCT04939610) was initiated to further evaluate the safety, dosimetry, and preliminary antitumor activity of [^177^Lu]Lu-FAP-2286 in patients with advanced solid tumors. In its phase I portion, 27 patients were recruited, with pancreatic (*n* = 9), colorectal (*n* = 4), and head and neck cancers (*n* = 3) being the most common tumor types (27). Treatment-related adverse events were observed in 14 patients with two experienced grade ≥ 3 events. dose-limiting toxicities included grade 4 lymphopenia at a dose of 5.55 GBq and grade 3 hemoptysis at 9.25 GBq. Importantly, no grade ≥ 3 reductions in neutrophil counts, hemoglobin levels, or platelet counts were noted. RECIST 1.1 evaluation revealed PR in one patient and SD in nine patients (27). Based these favourable safety and activity profiles, a phase II trial is underway to further evaluate the safety and efficacy of [^177^Lu]Lu-FAP-2286 as monotherapy and combination regimens with chemotherapy in selected solid tumors (NCT04939610).

More recently, two prospective studies further investigated the therapeutic potential of [^177^Lu]Lu-FAP-2286 (28, 29). In one study, five patients with advanced metastatic sarcoma received four cycles of [^177^Lu]Lu-FAP-2286 (doses ranging from 6,660 to 7,400 MBq) (28). Treatment was well tolerated, with no grade 3 or 4 toxicities reported. Quantitative imaging and volumetric analysis demonstrated a 52.37% mean reduction in primary tumor volume, along with substantial decreases in SUVmax and tumor-to-background ratio (TBR) of metastatic lesions (29.67% and 43.66%, respectively), particularly in pulmonary metastases. RECIST 1.1 assessment revealed PR in 4/5 (80%) patients and PD in 1/5 (20%) patient (28). Another study assessed safety and efficacy in nine patients with advanced lung cancer who received 2–6 cycles (7.4 GBq per cycle) of [^177^Lu]Lu-FAP-2286 (29). No grade 3 or 4 toxicities were observed throughout the follow-up period. Based on RECIST 1.1 and PERCIST 1.0 criteria, PR, SD, and PD were documented in 44%, 33.3%, and 22.2% of patients, respectively. The highest observed metabolic response reached a 66.89% reduction in tumor metabolic activity, and the ORR was 77.78%. Median OS and PFS were 10 and 6 months, respectively. The most significant improvements in clinical symptoms were noted in dyspnea and cancer-associated pain (29).

## 3BP-3940

As A novel derivative of FAP-2286, 3BP-3940 is another potential theranostic agent with improved pharmacokinetic properties, such as low renal uptake and longer tumor retention (30, 31). In a first-in-human clinical study, 3BP-3940 radiolabeled with ^177^Lu, ^90^Y, and ^225^Ac were injected in 88 patients with advanced metastatic malignancies of various histologies, including 21 pancreatic adenocarcinoma, 13 breast, 7 lung, and other types of cancer (32). The administration of [^177^Lu]Lu-/[^90^Y]Y-/[^225^Ac]Ac-3BP-3940 (with cumulative administered radioactivity per patient and range of ^177^Lu, 17.4 ± 13.0 GBq; ^90^Y, 6.9 ± 3.9 GBq; and ^225^Ac 13.4 ± 10.0 MBq) was in general well tolerated. Tumor response observed after any RLT cycle was 2/51 (3.9%) with CR, 32/51 (62.7%) with PR, 7/51 (13.7%) with SD, 8/51 (15.7%) with PD, and 2/51 (3.9%) with a mixed response resulting an ORR of 66.7% and a DCR of 80.4%. For the entire cohort, the median OS was 7.5 months from the start of RLT (32).

The results in subgroup of patients were also reported in detail by the same group. In a subgroup of 15 pancreatic ductal adenocarcinoma (PDAC) patients, a total of 34 cycles of [^225^Ac]Ac-3BP-3940 were administered over 40 months (5 monotherapies, 25 TANDEM ^225^Ac/^177^Lu, 4 TANDEM ^225^Ac/^90^Y) (33). The RLT was well tolerated with no long-term severe (G3/G4) hematological or renal toxicity documented. Therapy response evaluation (RECIST and THERCIST) showed PR in 4 patients (27%) and PD in 11 patients (73%). OS ranged from 2 to 14 months (median 6 months) (33). In breast cancer subgroup, 9 patients received a total of 17 cycles of [^225^Ac]Ac-3BP-3940 over 40 months (2 monotherapies, 13 TANDEM ^225^Ac/^177^Lu, 2 TANDEM ^225^Ac/^90^Y) with no cases of severe (G3/G4) nephrotoxicity observed (34). Therapy response assessment in 5 patients documented 2 PR (22%), 1 SD (11%) and 2 PD (22%). The remaining 4 patients experienced clinical progression leading to death. At the time of the analysis (late December 2024), 2 patients were alive (follow-up time of 7 and 20 months), and 7 patients had died (OS range 1–7 months, median 6 months) (34). Additionally, two cases with high-grade muscle invasive bladder cancer also showed remarkable response with [^177^Lu]Lu-3BP-3940 treatment (35). One male patient underwent two cycles of intravesical instillations of [^177^Lu]Lu-3BP-3940 (3.7 and 2.9 GBq), and the second with a concurrent systemic administration of [^177^Lu]Lu-3BP-3940 (3.9 GBq) and pembrolizumab as radiosensitizer (200 mg). This patient achieved PR (PET/CT 7 months later) and DFS of 22 months (surgery for bladder-intestine fistula confirmed the absence of residual tumor). Another female patient received two [^177^Lu]Lu-3BP-3940 intravesical instillations (5.4 and 4.8 GBq), the second paired with a systemic dose (7 GBq), achieving nearly complete remission two months later. No long-term hematological, renal or hepatic toxicity were reported (35).

### FAPI-RGD

The heterodimeric radioligand FAPI-RGD, which simultaneously targets FAP and integrin αvβ3 (RGD), has demonstrated superior tumor uptake and retention compared to its monomeric counterparts (36). In a first-in-human dosimetry study involving nine patients with advanced solid malignancies, administration of [^177^Lu]Lu-DOTA-FAPI-RGD was well tolerated, with no observed adverse clinical symptoms or pharmacological effects of concern (37). The whole-body effective radiation dose was estimated at 0.06 ± 0.03 Gy/GBq. The mean absorbed doses of red marrow, liver, spleen and kidneys were 0.04 ± 0.02 Gy/GBq, 0.56 ± 0.44 Gy/GBq, 0.28 ± 0.13 Gy/GBq, 0.34 ± 0.27 Gy/GBq, respectively. The agent exhibited substantial tumor localization and prolonged retention, resulting in high absorbed tumor doses 4.3 ± 0.7 Gy/GBq in pulmonary metastases and 4.0 ± 1.8 Gy/GBq in peritoneal metastases. According to RECIST criteria, PR was achieved in 2 patients (22.2%), SD in 6 patients (66.7%), and PD in 1 patient (11.1%). The ORR was 22.2%, and the DCR was 88.9% (37).

## Challenges and future perspectives

Despite encouraging preclinical data, not all FAP targeting RLT has successfully been translated into the clinic. For example, [^177^Lu]Lu-PNT6555 demonstrated prolonged tumor retention (up to 168 h) and notable antitumor efficacy at well-tolerated doses in HEK-mFAP xenograft mouse models (38). However, in a phase I dose-escalation clinical trial (NCT05432193) involving patients with various solid tumors, [^177^Lu]Lu-PNT6555 exhibited a low mean specific absorbed tumor dose of 0.16 Gy/GBq (range: 0.02–0.45 Gy/GBq), suggesting tumor retention time was limited, in sharp contrast to the observations in preclinical models (39). These suboptimal dosimetric outcomes necessitated a re-evaluation and further preclinical optimization to enhance the ligand's tumor-targeting efficiency and pharmacokinetic characteristics. Currently, additional FAP targeting radioligands are being developed, showing excellent preclinical antitumor activity, such as [^177^Lu]Lu-OncoFAP-23 (40), [^177^Lu]Lu-CTR-FAPI (41), [^177^Lu]Lu-NNS309 (42), [^177^Lu]Lu-FAP-HXN (43), [^177^Lu]Lu-DOTA-2P(FAPI)_2_ (44, 45), and [^177^Lu]Lu-JH04 (46). The safety and efficacy of these radioligands in clinical trials are pending.

For RLT, ideal FAP tracers should achieve intratumoral retention commensurate with the half-life of the therapeutic isotope to ensure effective tumor dosing and minimize systemic toxicity. However, current tracers fall short of this benchmark. For instance, the half-life of FAPI-46 (24 h) is shorter than that of ^90^Y (64 h), while FAP-2286 (44 h), DOTAGA(SA.FAPi)_2_ (86.6 h), and EB-FAPI (92.4 h) remain mismatched with longer-lived isotopes such as ^177^Lu (6.7 d) and ^225^Ac (9.9 d). Accordingly, FAPI-46 may be better suited to short-lived emitters such as ^213^Bi (46 min) or ^188^Re (17 h), whereas FAP-2286, DOTAGA(SA.FAPi)_2_, and EB-FAPI may pair more effectively with intermediate-lived radionuclides such as ^67^Cu (61.8 h) or ^90^Y. In addition, comprehensive patient-based dosimetry in large cohorts remains critical to accurately quantify tumor and normal tissue exposure, guide rational isotope selection, and optimize therapeutic indices for FAP-RLT.

In addition, the response of FAP-RLT in cancer patients should be interpreted with caution. FAP-RLT mainly targets FAP + CAFs, and direct cytotoxic effects on tumor cells are likely mediated only by β-emitting radionuclides (e.g., ^90^Y and ^177^Lu) through crossfire effects (47). Thus, observed clinical responses may largely reflect stromal depletion rather than direct tumor cell killing. The extent to which FAP-RLT exerts direct antitumor effects remains unresolved. Nevertheless, preclinical and clinical evidence supports the capacity of FAP-targeted therapy to remodel the tumor stroma, reduce physical barriers to drug delivery, and reprogram the immunosuppressive TME (48–53), thereby potentially enhancing the efficacy of established therapeutic modalities. The synergistic benefits of FAP-RLT combined with ICIs or cytotoxic chemotherapy have recently been indicated in some preclinical and clinical studies (45, 54–57). Combinatorial strategies integrating FAP-targeted therapy with other modalities, such as chemotherapy, immunotherapy, or tumor-directed agents are likely to achieve superior outcomes than either monotherapy. Future clinical trials are warranted to define the optimal patient populations and combinatorial strategies for FAP-targeted RLT.

## Conclusions

FAP-RLT is emerging as a promising pan-tumor targeted therapy. As a monotherapy, FAP-RLT is well tolerated with manageable side effects, showing encouraging response to tumors with high levels of FAP expression, such as sarcoma, PDAC, breast cancer, RR-DTC, and lung cancer. Except for sarcoma, FAP is mainly expressed on CAFs in most solid tumors. Therefore, the future direction would be to a combinatorial strategy, with FAP-RLT targeting the stiff stroma while other modalities targeting tumor themselves.
